# Ecological sustainability and high-quality development of the Yellow River Delta in China based on the improved ecological footprint model

**DOI:** 10.1038/s41598-023-30896-2

**Published:** 2023-03-07

**Authors:** Zhongyong Wei, Zhen Jian, Yingjun Sun, Fang Pan, Haifeng Han, Qinghao Liu, Yuang Mei

**Affiliations:** 1 School of Surveying and Geo-Informatics, Shandong Jianzhu University, Jinan, 250101 Shandong China; 2 Geophysical Prospecting and Surveying Team of Shandong Bureau of Coal Geological, Jinan, 250101 Shandong China; 3 Shandong Provincial Institute of Land Surveying and Mapping, Jinan, 250101 Shandong China

**Keywords:** Urban ecology, Environmental economics, Ecosystem services

## Abstract

Aiming at the traditional ecological footprint model, the improved ecological footprint of the carbon footprint effectively makes up for the singularity of the ecological footprint's consideration of carbon emissions, and plays an important role in promoting high-quality development and ecological sustainability. This paper selects 2015, 2018 and 2020 as important time points for the study, corrects the ecological footprint parameter factors based on net primary productivity (NPP), measures the ecological footprint after the improvement of the carbon footprint, studies the spatial and temporal variation in the ecological footprint at the 100-m grid scale with the support of IPCC greenhouse gas inventory analysis, and analyzes the current ecological conservation status of the Yellow River Delta. Additionally, in the context of a low carbon economy, the decoupling index of carbon emissions and GDP is extended to the evaluation and analysis of high-quality development. The study showed that (1) the ecological footprint of the Yellow River Delta has increased year by year, from 0.721 hm^2^·person^− 1^ to 0.758 hm^2^·person^− 1^, an average annual increase of 2.9%; the ecological carrying capacity has decreased from 0.40 hm^2^·person^− 1^ to 0.31 hm^2^·person^− 1^, an overall decrease of 28.59%. (2) The overall ecological deficit of the Yellow River Delta grid is lightly overloaded, with most of the ecological surplus occurring in the northern and eastern parts of the study area and a few moderate and heavy overloads in the center of the core area where there is a lot of built-up land and the area is small and easy to gather. (3) Based on the low-carbon economy analysis, 2015, 2017 and 2020 reach absolute decoupling and are in the ideal scenario. However, in the rest of the years, carbon emissions and economic development are still in a large contradiction, and decoupling has fluctuated and varied greatly in the last six years. The effective combination of ecological footprint and low carbon economy analysis provides an important theoretical basis for improving ecological conservation and achieving high-quality development.

## Introduction

The Yellow River Delta is rich in wetland resources and of great ecological value, with three world-class natural resources: river and sea confluence, nascent wetlands and wild birds, and is one of the first international wetland cities in China, undertaking a major historical mission to implement national strategies^1,2^. However, as the exploitation of oil resources, agricultural production, urban industry and other human activities are damaging the sustainability of the ecological environment to various degrees and affecting the level of quality development, it is of far-reaching significance to explore the ecological protection of the Yellow River Delta and evaluate high-quality development methods. Current methods of measuring sustainability include ecological footprint analysis, environmental sustainability index, comprehensive evaluation of indicator systems and energy value analysis^3–6^. The ecological footprint, also known as “ecological occupation”, is the sum of all resources consumed in an area, as well as the biological production land needed to absorb waste generated in the area^7^. First proposed by Canadian economists such as Rees^8^ in 1992 and further refined by Wackernagel^9^ in 1996, it has evolved from its initial concept and application to examine how to reduce the production of resources that human economies take from nature, and it provides an accounting framework for the biophysical services that a given economy needs to take from nature, thus providing a powerful tool for assessing sustainable development a powerful tool for assessing sustainable development. Sustainable development at the urban scale can be studied by analyzing the long-term changes in the ecological footprint at the local level^10^. The spatial effects of the ecological footprint index can also be analyzed through the impacts of neighboring countries to study sustainable development at the national scale^11^. The ecological footprint evaluation method has unparalleled applicability compared to other evaluation methods regardless of the spatial scale at which it is applied.

In the traditional ecological footprint assessment method, the parameter factors mostly refer to the research results of domestic and foreign scholars, but in recent years, the net primary productivity (NPP) of vegetation based on the remote sensing estimation method has been applied to improve the ecological footprint^12^. A number of studies have also emerged in China to improve the ecological footprint based on NPP^13–17^. The application of remote sensing products to the measurement of ecological footprint parameter factors can greatly improve the spatial and temporal sensitivity of the ecological footprint.

In the context of the new era of establishing a sound economic system of green, low-carbon and circular development, green development is an important symbol of China's shift from a speed economy to high-quality development. Since the 1970s, the global carbon cycle has been of concern to all sectors of society, particularly human activities that contribute to global warming and increased carbon emissions^18,19^. Land use carbon effects refer to the processes, activities and mechanisms by which carbon is released into the atmosphere from land that is influenced by human social and natural activities^20^. Carbon emissions from land use activities take into account not only CO_2_ from the combustion of fossil energy but also carbon emissions from other land use types. This paper, therefore, improves the fossil energy footprint of the traditional ecological footprint by using a carbon footprint that is closely linked to carbon emissions, which not only effectively addresses the single consideration of carbon emissions in the traditional ecological footprint, but also better reflects the trend of the carbon footprint based on carbon emission estimates in the total ecological footprint. Second, the causal relationship between environmental degradation caused by carbon emissions and economic growth can also be explored through various methods, such as simple regression, multiple cointegration and other linear regression methods^21^. However, among the many methods, the decoupling method, which relies on carbon emissions, is a precise means of studying the relationship between economic dependence on energy or greenhouse gases (atmospheric pollutants)^22^. The decoupling of economic growth from carbon emissions and energy consumption is a key indicator of green and high-quality development and an inevitable requirement for achieving carbon neutrality by 2060^23^. The methods commonly used to construct the decoupling index include the decoupling factor method, IPAT method, Kuznets method and Tapio decoupling elasticity coefficient method, among which the Tapio decoupling method integrates the changes of total and relative quantities to better fit the content of this study. Therefore, based on the improved ecological footprint of the carbon footprint and low-carbon economy, this paper evaluates the ecological protection status of the Yellow River Delta and promotes high-quality development under the green economy.

## Study area and data

### Study area

**Figure 1:**
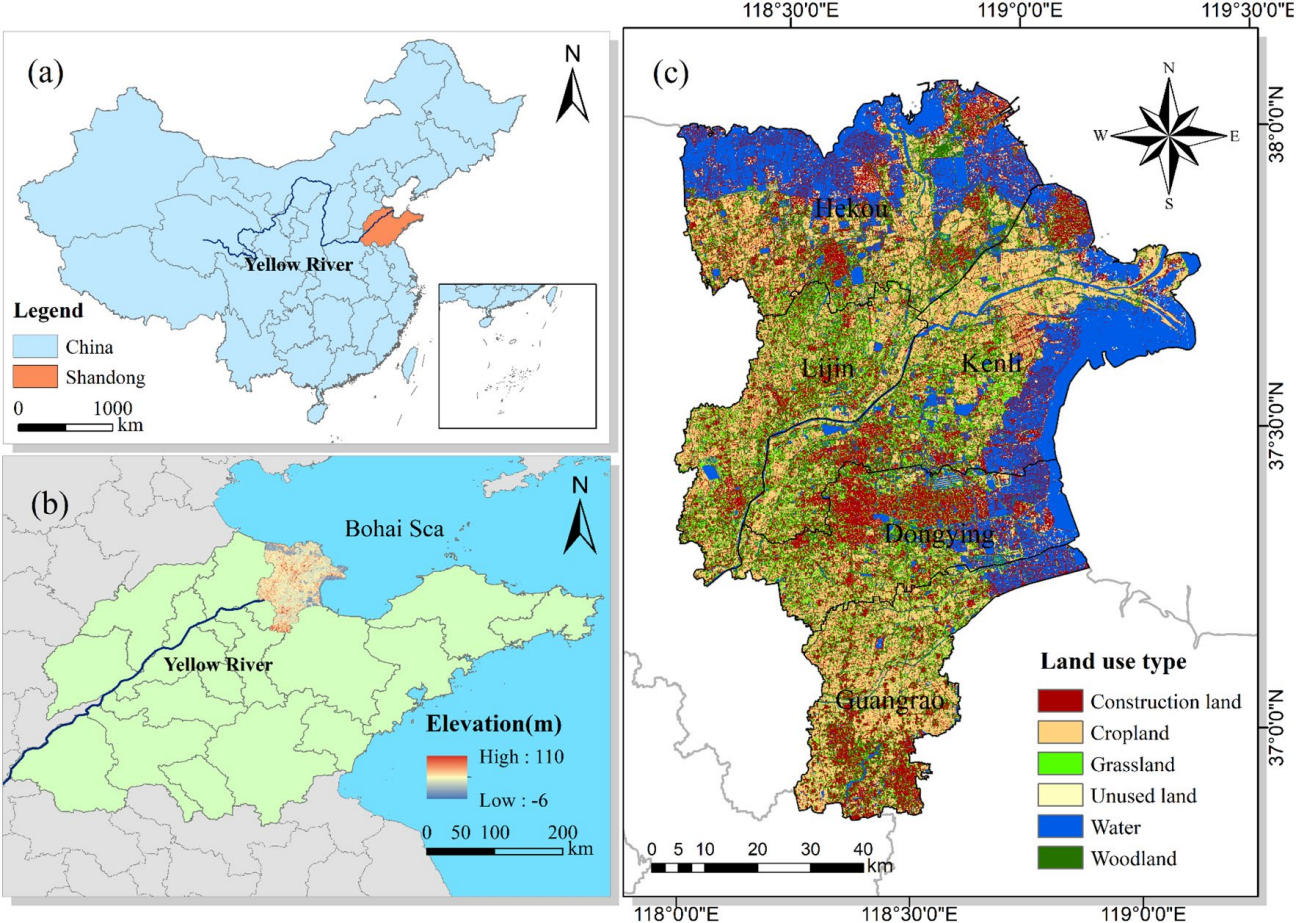
Geographical location of the study area and its counties (administrative divisions and names are from online open-source data). (**a**) Specific location of Shandong Province relative to China. (**b**) Specific location of the study area relative to Shandong Province and its elevation (m). (**c**) Land use distribution map of the study area with an accuracy of 30 m. Software: ArcGIS 10.2 (https://support.esri.com/en/download/2093).

Geographically, the definitions of the modern and contemporary Yellow River Delta are very different, as they refer to the Yellow River Delta with the town of Ninghai in Kenli County as the apex or the Yewwa as the apex, respectively, and the main area is located within the administrative limits of Dongying city, Shandong Province. For the purpose of this study, the study area of the Yellow River Delta referred to in this paper is within the city of Dongying, which has five county-level administrative units: Dongying District, Hekou District, Kenli District, Lijin County and Guangrao County (Fig. 1). Its special location at the mouth of the Yellow River has created a unique ecological environment, with a total wetland area of 4,580 km^2^, a wetland rate of 41.58%, and rich oil and gas resources. In 2020, Dongying's GDP was 2981.19 hundred million yuan, accounting for 4.08% of the province's GDP in the same period.

### Data and research framework

Land coverage data was based on Sentinel-2 multispectral imagery from the ESA Copernicus Data Centre (https://scihub.copernicus.eu/dhus/#/home), Manual visual interpretation through the eCongnition platform to generate land use raster datasets with a resolution of 30 m accuracy. MODIS' NPP product (MOD17A3H) is an annual data product from NASA (https://modis.gsfc.nasa.gov/) with a resolution of 500 m. The population density data were obtained from WorldPop (https://www.worldpop.org/), containing 1 km and 100 m precision, and the population density data of the 100 m × 100 m grid corrected by the United Nations were selected, taking into account the size of the study area, vegetation cover type and population mobility characteristics.

**Figure 2:**
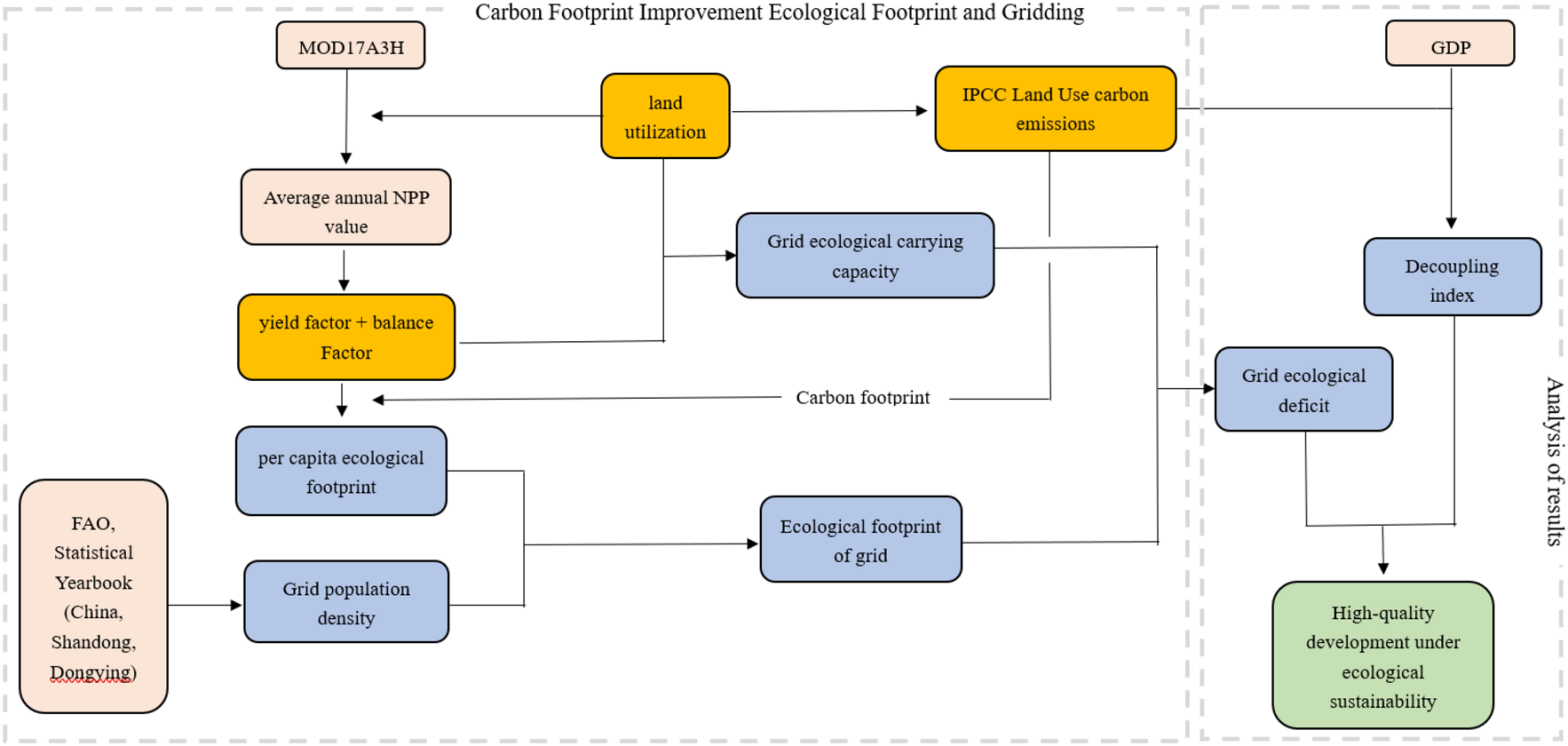
Ecological sustainability and high-quality development assessment process under grid ecological footprint and low-carbon economy.

Statistics are from the China Statistical Yearbook, Shandong Statistical Yearbook and Dongying Statistical Yearbook for 2015, 2018 and 2020. The world agricultural harvested area (farmed area), world average production and annual production are from the database of the Food and Agriculture Organization of the United Nations (FAO). World fish catch and aquaculture production are from the State of World Fisheries and Aquaculture 2020 published by FAO. The overall framework of this study is shown in Fig. 2.

## Methods

### Traditional ecological footprint consumption accounts

**Figure 3:**
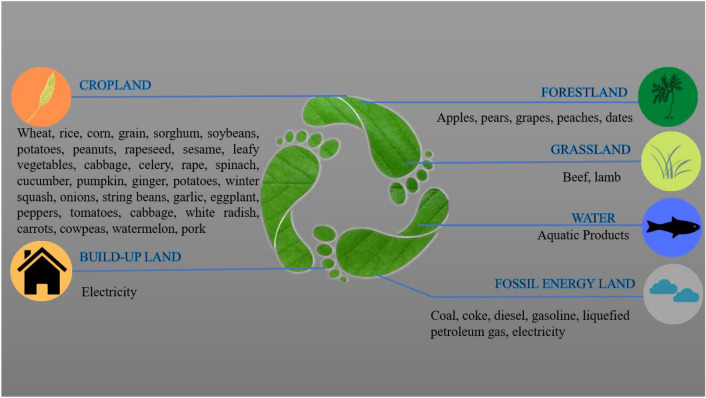
Traditional ecological footprint consumption accounts in Dongying city. This paper uses the carbon footprint to improve the fossil energy footprint of the traditional ecological footprint.

To truly reflect the ecological footprint and ecological carrying capacity of Dongying city, according to the lifestyle and consumption of Dongying city and with reference to Shandong Province Statistical Yearbook and Dongying City Statistical Yearbook, the biologically productive land is divided into arable land, forestland, grassland, water, construction land and fossil energy land, and the main consumption items of each category are shown in Fig. 3.

### NPP-based correction of ecological footprint parameters

The 30 m land use of the study area was resampled to 500 m, consistent with the resolution of MOD17A3H after pre-processing with MRT and other tools. Correction of ecological footprint parameter factors in Dongying City for 2015, 2018 and 2020 based on the annual average NPP of vegetation (Table 1). This method is faster and more accurate than other methods, and the implementation of NPP calculations from the vegetation light energy use efficiency (LUE) framework to correct ecological footprint parameters is more applicable and accurate than other methods.

**Table 1:** Average annual net primary productivity per land type in the Yellow River Delta.

Annual average NPP(kg)	2015	2018	2020
Cropland	0.345	0.375	0.403
Woodland	0.330	0.353	0.370
Grassland	0.307	0.329	0.342
waters	0.227	0.221	0.229
Construction land	0.280	0.298	0.315
Unused land	0.190	0.189	0.207

#### Yield factor

The formula for calculating the yield factor for arable land in the Yellow River Delta refers to NFA 2016:

1 $$\left\{ {\begin{array}{*{20}c} {Y_{j1} = \frac{{\Sigma A_{W} }}{{\Sigma A_{N} }}} \\ {A_{N} = \frac{{P_{N} }}{{Y_{N} }}} \\ {A_{W} = \frac{{P_{N} }}{{Y_{W} }}} \\ \end{array} } \right.$$

In Eq. (1), ${Y}_{j1}$ is the yield factor of the arable land in the study area, ${A}_{N}$ is the harvested area ( culture area ) of agricultural products of category $N$ in the study area, ${A}_{W}$ is the area required to produce an equivalent amount of this type of agricultural product based on the world average yield, ${P}_{N}$ is the production of agricultural products of category $N$ under the region, ${Y}_{N}$ is the average yield of agricultural products of category $N$ under the region, and ${Y}_{W}$ is the world average production of a category of agricultural products.

The NPP products from MODIS supported by remote sensing were used as the base data to correct the yield factors of woodlands and grasslands in the study area under the ecological footprint model.

2 $$Y_{{{\text{j}}2}} = \overline{{NPP_{local} }} /\overline{{NPP_{global} }}$$

In Eq. (2), ${Y}_{\mathrm{j}2}$ is the yield factor for woodland and grassland in the study area, ${NPP}_{local}$ is the average annual net primary productivity of woodland and grassland in the study area in the corresponding year, and ${NPP}_{global}$ is the global average NPP of woodland and grassland in the corresponding year, referring to Amthor et al.^24^.

In addition, most of the land for construction comes from cropland, so the yield factor for construction land is the same as that for cropland^25^. The yield factors for the watershed were derived from the Wackernagel and Rees^26^ study.

#### Balancing factor

The NPP model for provincial hectares was applied to the municipal scale. Among them, the NPP of four biologically productive lands, namely cropland, woodland, grassland and water, was weighted and summed to obtain the annual average NPP within the city area.

3 $$\overline{NPP} = \frac{{\mathop \sum \nolimits_{j} \left( {A_{j} \times NPP_{j} } \right)}}{{\mathop \sum \nolimits_{j} A_{j} }}$$

In Eq. (3), $\overline{NPP }$ is the average net primary productivity of arable land, forestland, grassland and water in Dongying, ${A}_{j}$ is the area of land in category $j$, and ${NPP}_{j}$ is the average annual NPP of productive land in category $j$.

Balancing factors for arable land, woodland, grassland and water in the Yellow River Delta.

4 $$R_{j} = \frac{{NPP_{j} }}{{\overline{NPP} }}$$

In Eq. (4), ${R}_{j}$ is a balancing factor.

The sites for construction are located in areas suitable for agricultural cultivation or directly occupy arable land, so the potential ecological productivity of urban construction land is the same as that of arable land, and therefore the equilibrium factor for construction land is equal to that of arable land^27^.

### Ecological footprint principles and improvements

#### Ecological footprint model

Ecological footprint model includes ecological footprint, ecological carrying capacity and ecological deficit. As the study area is within the city limits and the statistics have their own characteristics, adjustments have been made to the methodology for calculating the national ecological footprint accounts^28^. Based on the biological consumption account, the ecological footprint can be calculated for any land use type.

5 $$EF = \frac{P}{{Y_{N} }} \times R_{j} \times Y_{j}$$

In Eq. (5), $P$ is the number of biologically productive land harvesting consumption items in a category, and ${Y}_{N}$ is the average production of consumption Item $N$ in the region. The ecological footprint of the construction land is measured based on the area of human infrastructure land and is equal to its ecological carrying capacity.

Ecological carrying capacity is the determination of the maximum carrying capacity of an ecosystem for human activity, expressed as the sum of the biologically productive land area available in an area.

6 $$EC = N \times ec = N \times \sum \left( {a_{j} \times R_{j} \times Y_{j} } \right)$$

In Eq. (6), $EC$ is the ecological carrying capacity per capita, and ${a}_{j}$ is the per capita area of biologically productive land of category j in the region. According to the recommendations of the World Commission on Environment and Development, 12% of the ecological carrying capacity should also be deducted for biodiversity conservation. The population figures for the study area were obtained from the statistical yearbook and the seventh national census data. According to the recommendations of the World Commission on Environment and Development, 12% of the ecological carrying capacity should also be deducted for biodiversity conservation.

An ecological deficit is the interpolation of the ecological footprint and ecological carrying capacity.

7 $$ED = EF - EC$$

When $ED>0$ indicates an ecological deficit, the ecological environment has exceeded the carrying capacity. Conversely, when $ED<0$, the ecology of the study area is in surplus.

#### Land use carbon emissions

Based on the research of domestic and foreign scholars, this paper divides the carbon emission calculation of land use into a direct calculation method and an indirect calculation method, in which arable land, grassland, forestland, water area and garden land are the direct sources of carbon emissions, so the direct calculation method of carbon emissions is used; construction land is the indirect source of carbon emissions, so the indirect calculation is based on the carbon emissions generated after the fossil energy consumed by construction land.

(1) Direct calculation of carbon emissions.

Carbon emissions from arable land, forestland, grassland, water, garden land and unused land are non-building land, and their carbon emissions mainly come from the energy consumption of agricultural machinery, fertilizer application, biological respiration and decomposition of soil organic matter^29^, so they are calculated using the direct carbon emission calculation method.

8 $$C = \mathop \sum \limits_{i = 1}^{n} T_{i} = \mathop \sum \limits_{i = 1}^{n} e_{i} \times \delta_{i}$$

In Eq. (8), $C$ is the total carbon emissions of a site category, ${T}_{i}$ is the carbon emissions from land type $i$, ${e}_{i}$ denotes the area of land in category $i$, ${\delta }_{i}$ is the carbon emission factor (carbon sequestration factor) for land type $i$, Carbon emission is positive and carbon sink is negative. As shown in Table 2.

**Table 2:** Carbon emission estimation coefficient of nonconstruction land in the Yellow River Delta.

Land class	Carbon emission (absorption) Factors/(kg C/(hm^2^·a))	Reference sources
Cropland	422	Sun et al.^30^,Sun Hebin^31^
Woodland	− 644	Shi et al.^32^,Fang et al.^33^,Wang et al. ^31^
Grassland	− 21	Sun et al.^30^,Shi et al.^32^
Water	− 253	Sun et al.^30^,Shi et al.^32^
Garden	− 730	Fan et al.^34^
Unused land	5	Sun et al.^30^,Shi et al.^32^

(2) Indirect calculation of carbon emissions.

Since the calculation of carbon emissions from construction land is not suitable for direct estimation, the method of indirect estimation by constructing a carbon emission model for energy consumption is adopted^35^. The main types of energy consumed in the Yellow River Delta are coal, coke, crude oil, fuel oil, gasoline, and paraffin.

9 $$E = \sum T_{i} \times \alpha_{i} \times \beta_{i}$$

In Eq. (9), $E$ stands for total carbon emissions from fossil energy combustion, ${T}_{i}$ denotes the total consumption of fossil energy in category $i$, ${\alpha }_{i}$ is the coefficient of conversion of category $i$ fuel consumption into standard coal, and ${\beta }_{i}$ is the carbon emission conversion factor when type $i$ energy is consumed. As shown in Table 3.

**Table 3:** Carbon Emission Estimation Coefficient of Construction Land in Yellow River Delta.

Energy category	Discount factor for standard coal(kg cd/kg)	Carbon emission factor (t C/t)
Coal	0.714	0.756
Coke	0.971	0.855
Crude Oil	1.429	0.586
Fuel oil	1.429	0.619
Petrol	1.471	0.554
Paraffin	1.471	0.571
Diesel	1.457	0.592
Liquefied Petroleum Gas	1.714	0.504

#### Improvement ecological footprint based on carbon footprint

The ecological footprint of energy land reflects the degree of pressure on the surrounding ecological environment caused by the consumption of fossil fuels by human activities and economic development. The traditional method of measuring the ecological footprint of energy land mainly considers the CO_2_ emitted after the combustion of fossil energy. This paper takes into account the difference in carbon emissions during the land use process, based on the traditional ecological footprint consumption account, and replaces the traditional ecological footprint of energy land with a carbon footprint, which can better reflect the change pattern of carbon emissions in the total ecological footprint during human activities and is closely integrated with the IPCC land use carbon emissions study. It is also possible to take into account the impact of carbon emission factors on the carbon sequestered land in the ecological footprint.

10 $$EF_{C} = \frac{{\left( {E_{g} + E_{j} + E_{w} } \right)}}{NP}$$

In Eq. (10), ${EF}_{C}$ is the carbon footprint, ${E}_{g}$, ${E}_{j}$ and ${E}_{w}$ denote the total annual CO_2_ emissions from cropland, construction land and unused land respectively, and $NP$ is the average carbon sequestration capacity of grasslands, woodlands, gardens and watersheds, t/hm2.

#### Gridded ecological footprint model

While traditional ecological footprint estimation often takes administrative districts as the basic unit, the grid ecological footprint can show the spatial distribution of the ecological footprint within the study area at a large scale, free from the limitations of administrative units, and this method is more intuitive and accurate.

11 $$Ef_{j} = \frac{{EF_{i} }}{{P_{i} }} \times p_{j}$$

In Eq. (11), ${Ef}_{j}$ indicates the ecological footprint of the grid, ${EF}_{i}$ and ${P}_{i}$ denote the total ecological footprint and total population of the ith city respectively, and ${p}_{j}$ is the population density of grid $j$.

12 $$Ec_{j} = \sum R_{j} \times Y_{j} \times a_{ij}$$

13 $$Ed_{j} = Ef_{j} - Ec_{j}$$

In Eqs. (12) and (13), ${Ec}_{j}$ denotes the ecological carrying capacity of the grid, ${a}_{ji}$ denotes the area of productive land of category $i$ in grid $j$, and ${Ed}_{j}$ denotes the grid ecological deficit.

### Decoupling carbon emissions from economic growth

**Figure 4:**
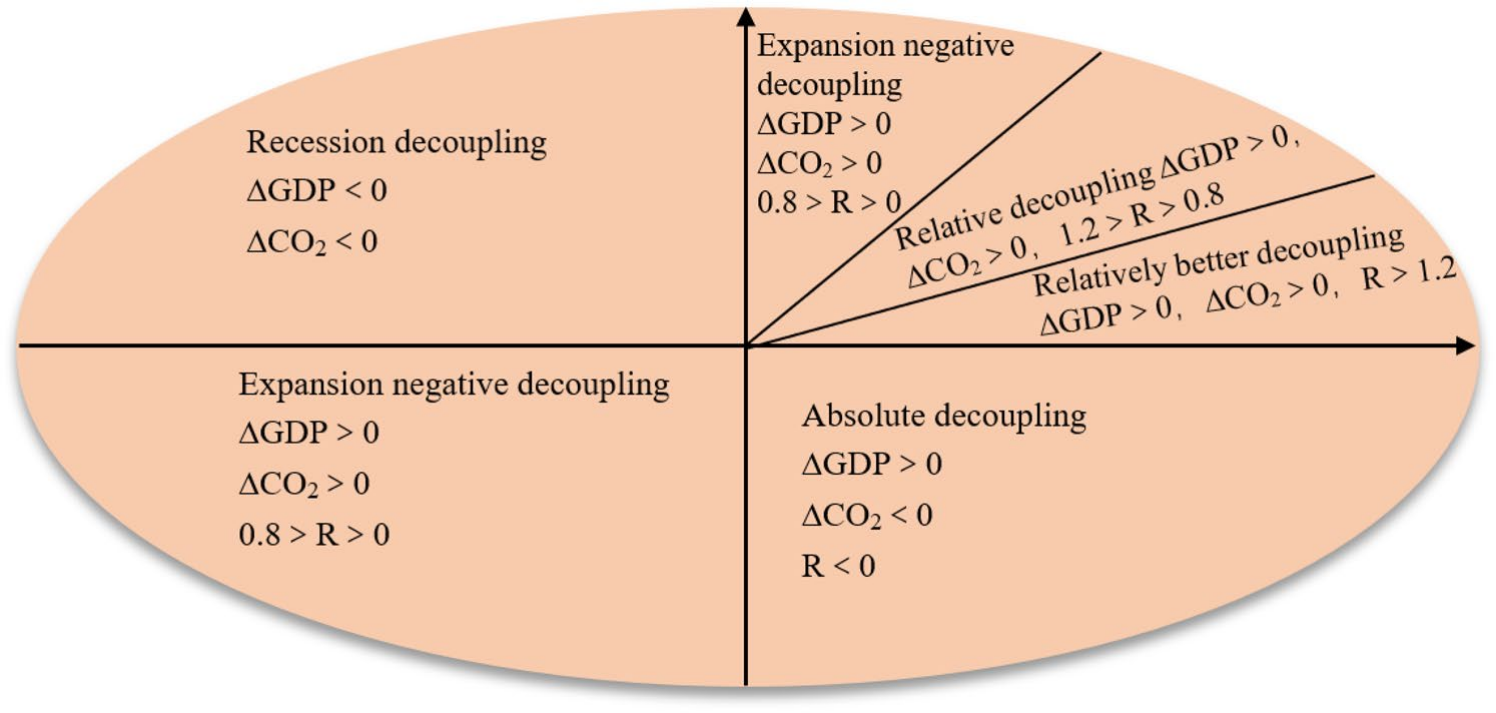
Types of decoupling of economic development and carbon emissions.

To develop a sound green economic system and empower China to 'double carbon', it is necessary to strengthen the management of carbon in the process of economic development and improve energy-based economic growth. Therefore, this paper introduces the decoupling relationship between carbon emissions and economic growth, and uses the three indicators of economic carbon emission factors $R$, $\Delta GDP$ and $\Delta {CO}_{2}$ as the basis for judging the degree of decoupling between carbon emissions and $GDP$(Fig. 4), which is of great practical significance for formulating reasonable low-carbon emission reduction plans, reducing ecological pressure and promoting high-quality development.

Tapio constructed the decoupling elasticity coefficient by calculating the ratio of the change in environmental pressure to the change in total economic volume. Based on this, this paper constructs a decoupling elasticity coefficient between carbon emissions and economic growth as a way to portray the synergistic relationship between carbon and the economy in the process of vigorous economic development in the Yellow River Delta (Table 4).

14 $$R = \frac{{\left( {C^{i} - C^{i - 1} } \right)/C^{i - 1} }}{{\left( {GDP^{i} - GDP^{i - 1} } \right)/GDP^{i - 1} }}$$

**Table 4:** The significance of decoupling economic development from carbon emissions.

Type of decoupling	Level	Status	Significance	Trends
Absolute decoupling	VI	Ideal	$\Delta GDP$ growing rapidly, $\Delta {CO}_{2}$ growing negatively. Energy utilization at its highest	
Relatively good decoupling	V	More desirable	Rapid growth in $\Delta GDP$ and $\Delta {CO}_{2}$, $\Delta GDP>\Delta {CO}_{2}$, high energy use efficiency	
Relative decoupling	IV	General	$\Delta GDP$ and $\Delta {CO}_{2}$ are growing rapidly at the same time, but resource utilization is not improving	
Expansion negative decoupling	III	more negative	$\Delta GDP$ and $\Delta {CO}_{2}$ increase, $\Delta GDP<\Delta {CO}_{2}$, carbon emissions increase, resource utilization decreases, ecological pressure increases	
Decline decoupling	II	Negative	Declining $GDP$ and lower ecological pressure	
Negative decoupling	I	Most negative	Declining $GDP$ and increasing ecological pressure	

Trends in $GDP$, Trends in carbon emissions, Trends in ecological pressures, Resource utili-zation.

In Eq. (14), ${C}^{i}$ denotes the carbon emissions in year $i$, ${C}^{i-1}$ denotes the carbon emissions in year $i-1$,and ${GDP}^{i}$ and ${GDP}^{i-1}$ denote the total economic output in years $i$ and $i-1$, respectively.

## Results

### Ecological footprint parameters

The ecological footprint yield factor and balance factor of the Yellow River Delta for 2015, 2018 and 2020 were calculated according to the above method and analyzed in comparison with the common global or Chinese common factor calculation results published by Wackernagel^26^, Liu Moucheng et.al.^13,36^ and WWF (https://www.wwfchina.org) at domestic and overseas(Table 5). Significant differences were found between the various land ecological footprint factors calculated based on different models and the results of this study. The output capacity of forest land is close to the global average, the balance factor of arable land and construction land is significantly lower than the common factor, and the watershed and grassland are higher than the common factor. It shows that the study area has a strong output capacity of cropland, grassland and water in 2015, 2018 and 2020, but the cropland is much lower than the average of global scale and Chinese scale, and only reflects the strongest output capacity within the study area. In terms of yield factors, the results calculated in this study were generally close to the common factors, with strong variability in yield factors across categories, including relatively high productivity in grassland.

**Table 5:** Yield factor and balance factor of each land type in Yellow River Delta.

Land class	cropland		Woodland		Grassland		Water		Construction land	
Year/Reference	${R}_{j}$	${Y}_{j}$	${R}_{j}$	${Y}_{j}$	${R}_{j}$	${Y}_{j}$	${R}_{j}$	${Y}_{j}$	${R}_{j}$	${Y}_{j}$
2015	1.15	1.74	1.10	0.47	1.03	0.88	0.76	1.00	1.15	1.74
2018	1.19	1.42	1.12	0.50	1.04	0.94	0.70	1.00	1.19	1.42
2020	1.24	1.33	1.14	0.53	1.05	0.98	0.70	1.00	1.24	1.33
Wackernagel^26^	2.80	1.66	1.10	0.91	0.5	0.19	0.20	1.00	2.80	1.66
WWF2004^37^	2.19	–	1.38	–	0.48	–	0.36	–	2.19	–
WWF2008^38^	2.39	–	1.25	–	0.51	–	0.41	–	2.39	–
Liu et al.^36^	1.74	1.74	1.41	0.86	0.44	0.51	0.35	0.74	1.74	1.74

The main reasons for this are the following factors that contribute to the unique ecological footprint parameter characteristics of the study area: (1) Productivity of various types of biologically productive land in the Yellow River Delta differs from the global average productivity. (2) Differences in model calculation methods. (3) The conservation of forests and grasslands in the study area has achieved remarkable results. (4) The special geographical location makes the study area rich in wetland resources with many lakes and waters.

### Carbon emissions

**Figure 5:**
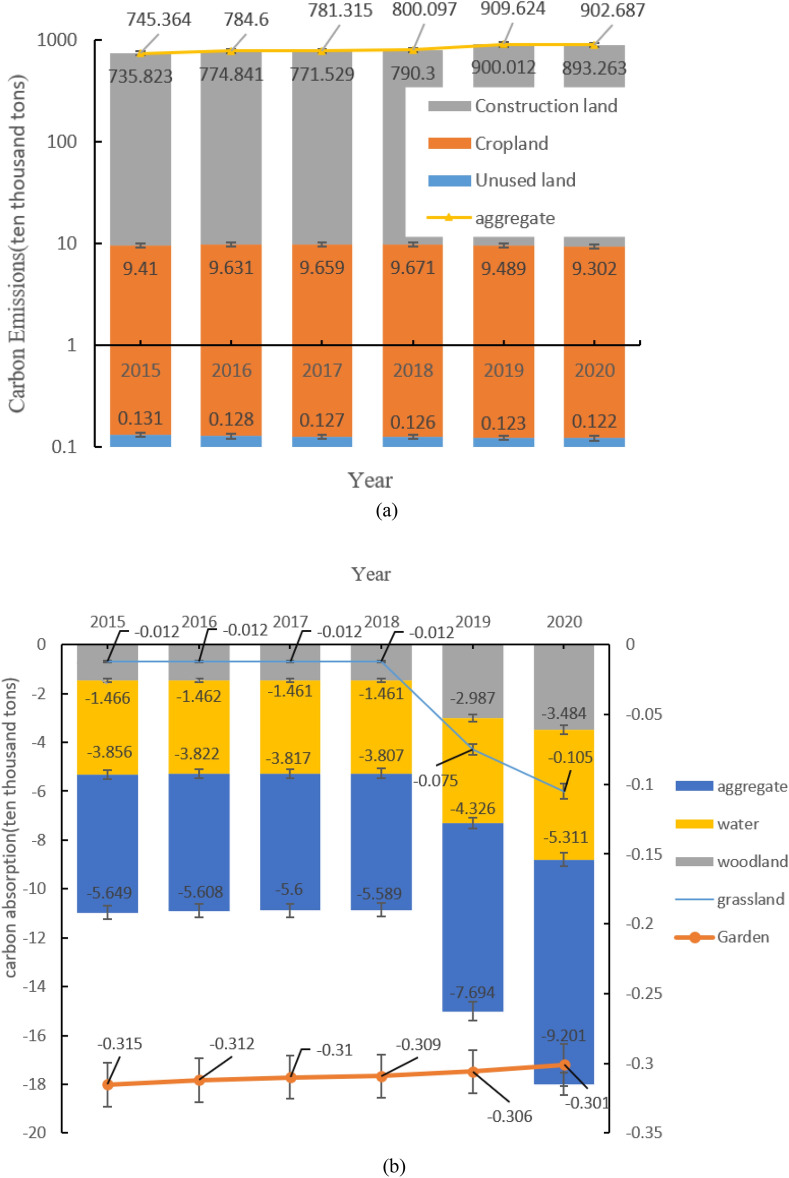
(**a**) Interannual trends in total carbon emissions from cropland, construction land and unused land. (**b**) Interannual trends in total carbon sequestration in grasslands, woodlands, gardens and watersheds.

As shown in Fig. 5 and Table 6, the Yellow River Delta land use carbon emissions are divided into two rising phases. The first stage is from 2015–2018, with carbon emissions increasing by 54.733 ten thousand tons over three years, with an average annual increase of 18.244 ten thousand tons, where carbon sinks decrease by approximately 0.06 ten thousand tons; the second stage is from 2018–2020, with carbon emissions increasing by 102.59 ten thousand tons over two years, with an average annual increase of 51.295 ten thousand tons, an increase of 3.612 ten thousand tons in carbon sinks, a significant increase compared to the first stage of carbon emissions, the growth is about twice as much as the first stage. The trend of carbon emissions from cropland shows a “V” shape; construction land is the main source of carbon emissions, accounting for more than 98% of the total carbon emissions in the same year; carbon sequestration mainly relies on woodland and water, with woodland and water accounting for more than 94% of the total carbon sequestration in the same year. Overall, carbon emissions in the study area are much greater than carbon sinks.

**Table 6:** Estimates of carbon emissions from land use in the Yellow River Delta 2015–2020 (ten thousand tons).

	Carbon emissions	2015	2016	2017	2018	2019	2020
Carbon Source	Cropland	9.410	9.631	9.659	9.671	9.489	9.302
	Construction land	735.823	774.841	771.529	790.300	900.012	893.263
	Unused land	0.131	0.128	0.127	0.126	0.123	0.122
	Total	745.364	784.600	781.315	800.097	909.624	902.687
Carbon sinks	Grassland	− 0.012	− 0.012	− 0.012	− 0.012	− 0.075	− 0.105
	Woodland	− 1.466	− 1.462	− 1.461	− 1.461	− 2.987	− 3.484
	Garden	− 0.315	− 0.312	− 0.310	− 0.309	− 0.306	− 0.301
	Water	− 3.856	− 3.822	− 3.817	− 3.807	− 4.326	− 5.311
	Total	− 5.649	− 5.608	− 5.600	− 5.589	− 7.694	− 9.201
Net carbon emissions	739.715	778.992	775.715	794.508	901.93	893.486	

### Ecological footprint

**Figure 6:**
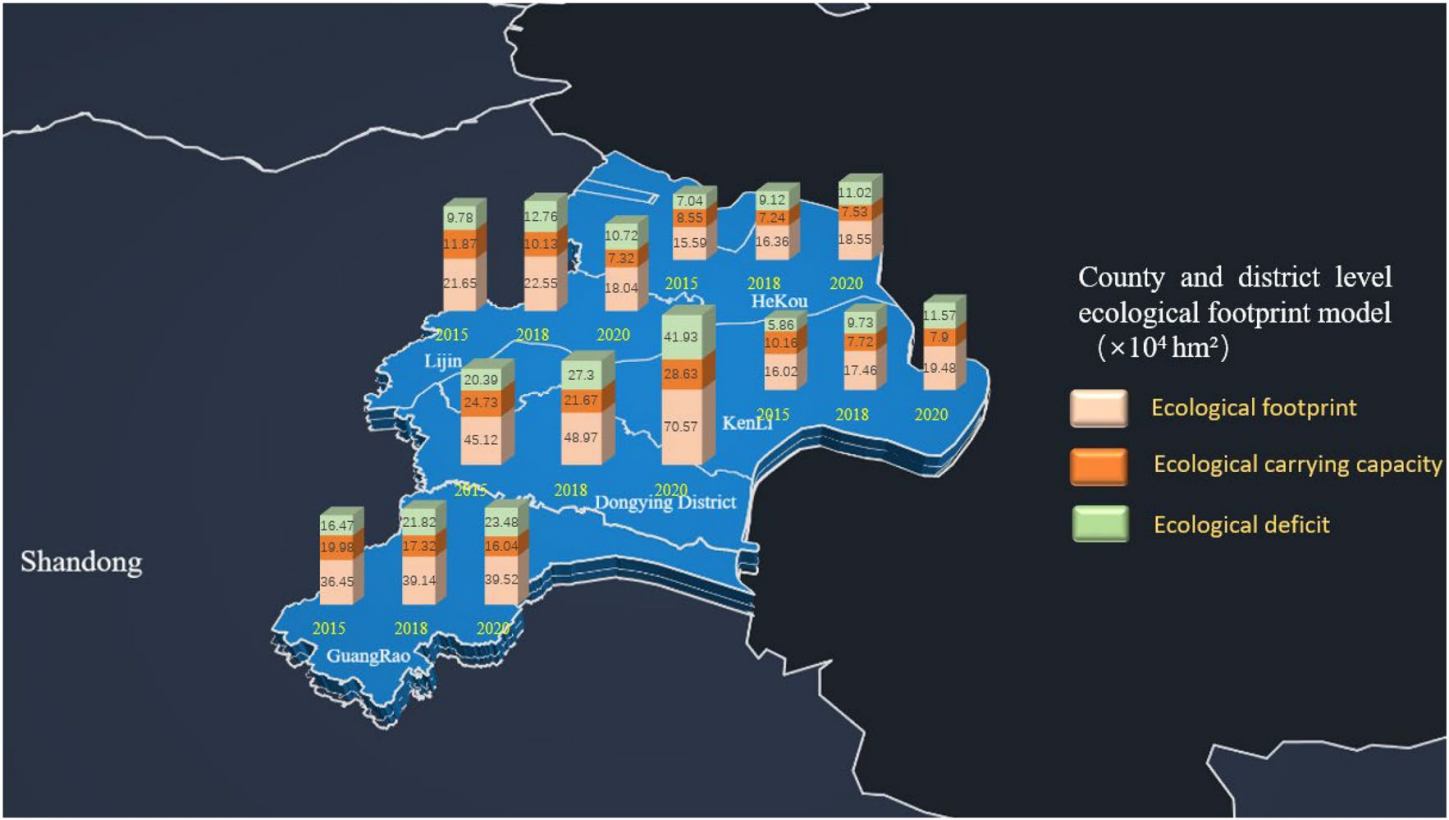
Total ecological footprint model for each county. This figure was created with Photoshop 2020 (https://www.adobe.com).

The ecological footprint per capita is increasing year by year, with an average annual growth rate of approximately 2.9%, among which the ecological footprint of Dongying District is significantly higher than that of other counties and districts, occupying 33.46% of the total ecological footprint of Dongying City; the ecological carrying capacity is decreasing year by year, with a decrease of approximately 28.59%, among which the carbon footprint occupies the primary part of approximately 28.28%; the ecological deficit is increasing year by year, Dongying District, the largest increase in ecological deficit in 2020, two times in 2015, a sharp increase in ecological pressure. The results were given in Fig. 6 and Table 7.

**Table 7:** Ecological footprint model per capita in the Yellow River Delta (hm^2^/person). Average ecological carrying capacity per capita in China from data published by the Global Footprint Network (https://www.footprintnetwork.org/).

Year	2015			2018			2020		
Land type	EF	EC	ED	EF	EC	ED	EF	EC	ED
Cropland	0.1949	0.2030	− 0.0151	0.2330	0.1748	− 0.0582	0.2013	0.1458	0.0555
Woodland	0.0021	0.0055	− 0.0034	0.0020	0.0057	0.0582	0.0019	0.0134	− 0.0112
Grassland	0.0003	0.0024	− 0.0021	0.0004	0.0025	− 0.0037	0.0005	0.0207	− 0.0202
Water	0.1714	0.0467	0.2470	0.1428	0.0446	0.0982	0.1744	0.0319	0.1425
Construction land	0.1485	0.1307	0.0178	0.1232	0.1010	0.0222	0.1090	0.0959	0.0131
Carbon footprint	0.2039	0	0.2039	0.2412	0	0.2412	0.2070	0	0.2704
Total	0.7211	0.3953	0.3258	0.7426	0.3074	0.4140	0.7575	0.3074	0.4501
China	–	0.9	–	–	0.9	–	–	0.9	–

### Ecological footprint model at the grid scale

#### Spatial heterogeneity of ecological footprints

**Figure 7:**
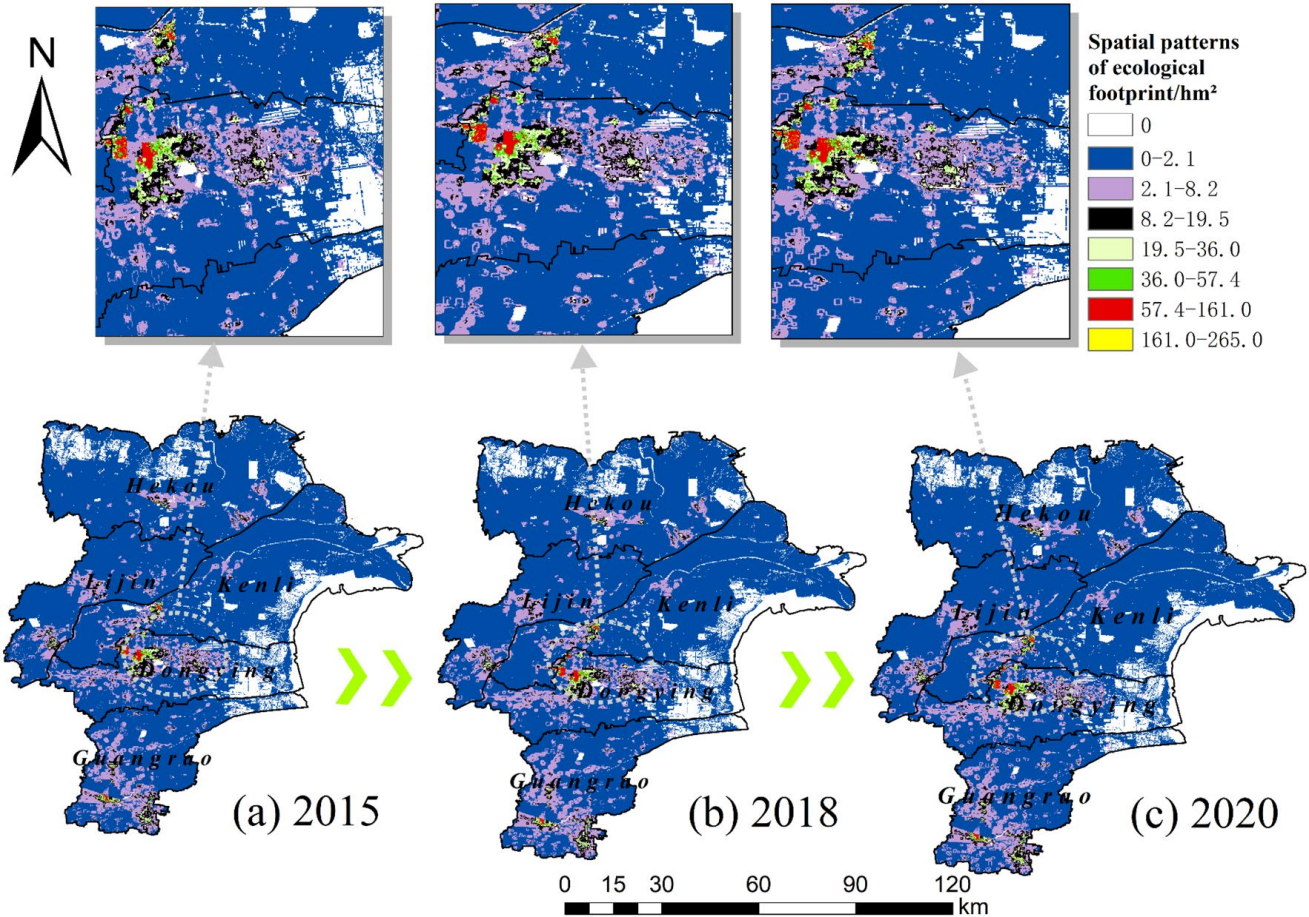
Spatial heterogeneity of ecological footprints in 2015 (**a**), 2018 (**b**) and 2020 (**c**). This figure was created with ArcGIS 10.2 (https://support.esri.com/en/download/2093).

ArcGIS provides powerful spatial analysis functions, and through gridding, the spatial heterogeneity of the ecological footprint can be clearly expressed. The areas with high ecological footprints are distributed in the main urban areas of Dongying city and are increasing year by year and spreading out from the center of the county (Fig. 7). Dongying City is a coastal city and is in the special position of the Yellow River estuary, which makes Dongying City more inland Wetlands by the sea and less floating population in the region, making the ecological footprint of some areas within the urban fringe approximate to zero. Dongying City has a wide distribution of cultivated land and strong population aggregation. Therefore, the ecological footprint is mostly concentrated and distributed in Dongying District and Guangrao County. Among them, the ecological footprint of Dongying District is as high as 33.46% of the total ecological footprint of the study area, and the pressure on the ecology exceeds the rest of the counties.

#### Spatial Heterogeneity of Ecological Carrying Capacity

**Figure 8:**
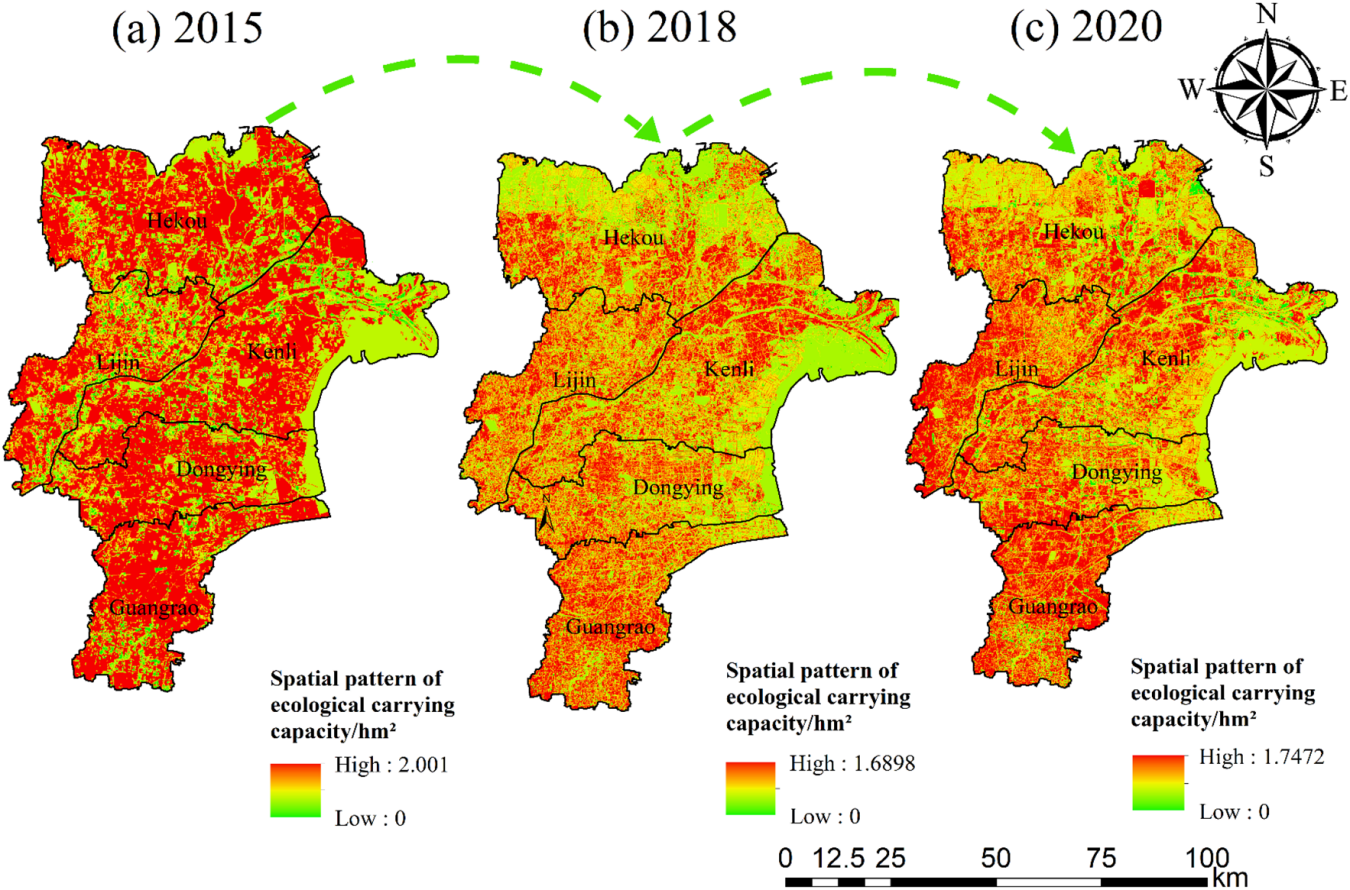
Spatial heterogeneity of ecological carrying capacity in 2015 (**a**), 2018 (**b**) and 2020 (**c**). This figure was created with ArcGIS 10.2 (https://support.esri.com/en/download/2093).

The ecological carrying capacity peaked at 2.001 hm^2^ in 2015 and then began to decline until 2018, when it began to rise (Fig. 8). This result demonstrates that wetlands and grasslands are being protected and arable land is being improved in this area during this period.

#### Spatial heterogeneity of ecological deficit

**Figure 9:**
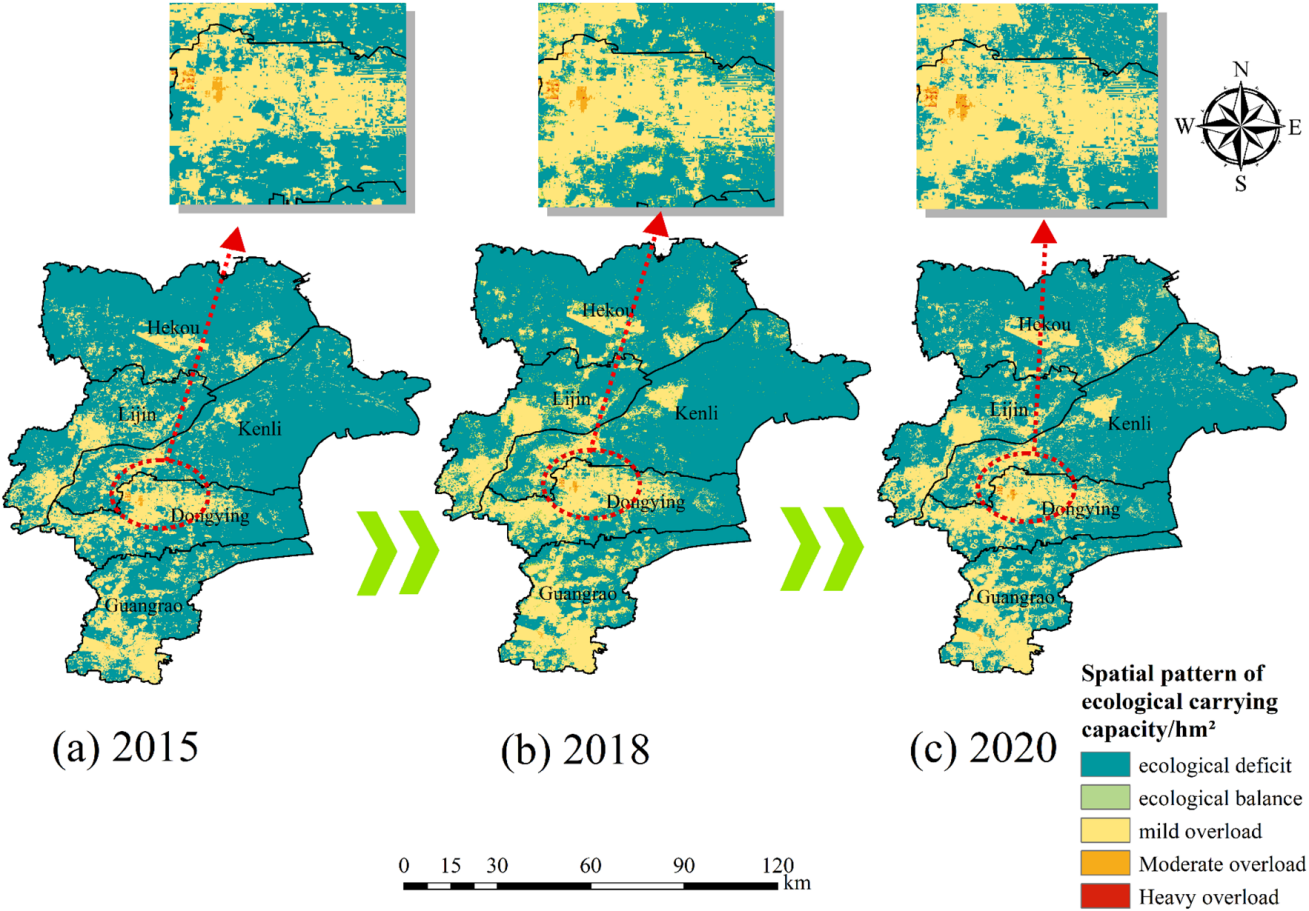
Spatial heterogeneity of ecological deficits in 2015 (**a**), 2018 (**b**) and 2020 (**c**). This figure was created with ArcGIS 10.2 (https://support.esri.com/en/download/2093).

To visually reflect the ecological pressure distribution, the ecological surplus and deficit of the grid are divided according to a certain range. The original model considers the ecological deficit to be in ecological equilibrium when it is equal to zero, in overload when it is greater than zero, and in surplus when it is less than zero. Meanwhile, according to the actual situation of the Yellow River Delta, the overload state is divided into three categories (light overload, moderate overload and heavy overload) by applying the natural breakpoint method to the overload results, as shown in Fig. 9.

### Decoupling carbon emissions from economic growth

**Figure 10:**
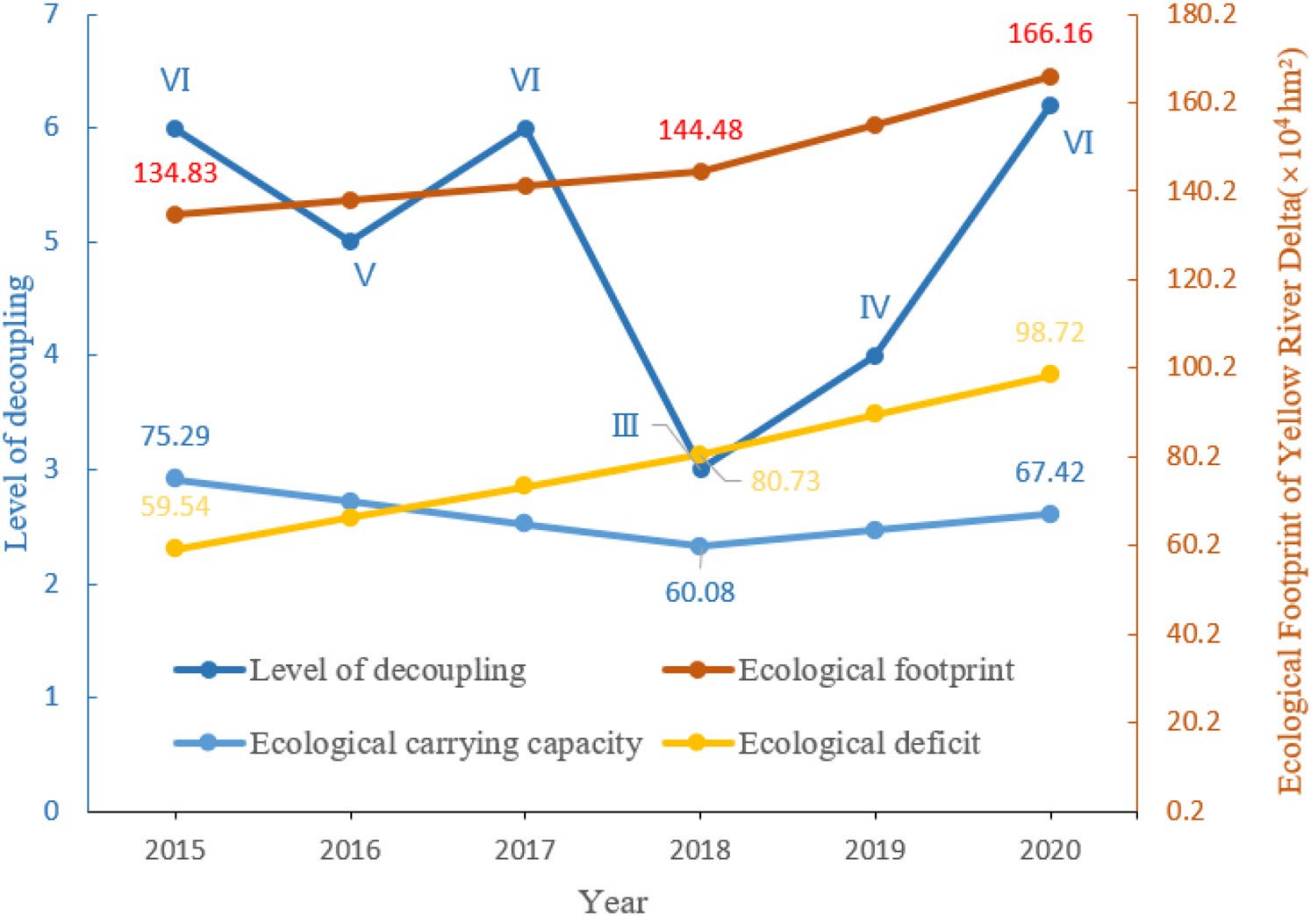
Ecological footprint and decoupling time series changes.

The decoupling index of the Yellow River Delta from 2015 to 2020 was estimated based on the elasticity coefficient of carbon emissions and economic growth constructed by Tapio decoupling theory (Table 8). The results show that the absolute decoupling state is reached in 2015, 2017 and 2020, and with rapid economic development, carbon emissions decrease accordingly, while the economy and carbon emissions remain in a large contradiction in the rest of the years, and the decoupling state shows significant irregular fluctuations with large variability over five years. In contrast, the ecological footprint of the same period increased year by year and reached the peak of the study in 2020. The ecological carrying capacity decreased overall, and the ecological deficit increased year by year and exceeded the growth rate of the ecological footprint (Fig. 10).

**Table 8:** Carbon Emissions and Economic Decoupling Index for the Yellow River Delta 2015–2020 ($GDP$: hundred million yuan, ${CO}_{2}$: ten thousand tons).

Year	$\Delta GDP$	$\Delta {CO}_{2}$	$R$	Decoupling status
2015	200.440	− 27.427	− 0.575	Absolute decoupling
2016	32.350	39.236	1.213	Relatively good decoupling
2017	206.060	− 3.285	− 0.016	Absolute decoupling
2018	149.590	18.782	0.126	Expansion negative decoupling
2019	131.380	109.527	0.837	Relative decoupling
2020	60.000	− 6.937	− 0.107	Absolute decoupling

## Conclusion and discussion

### Discussion

The traditional method of measuring the ecological footprint mainly takes into account CO_2_ emissions from fossil energy combustion, ignoring the difference in carbon emissions during land use. Improving the ecological footprint with the carbon footprint can better reflect the change pattern of carbon emissions in the total ecological footprint during human activities, while the introduction of a decoupling index provides data support for promoting high-quality development in the context of a low-carbon economy. Based on the above methods, this study uses the improved ecological footprint as an important tool to evaluate sustainable development, by the degree of utilization of ecological resources and the development trend of multi-year deficit, reflecting the sustainable development of the study area, and the decoupling index to evaluate the synergistic relationship between carbon emissions and economic development, reflecting the high-quality development in the context of low-carbon economy. The results show that the study area was in ecological deficit in 2015, 2018 and 2020, in a state of unsustainable development and worsening every year, and reached absolute decoupling in 2015, 2017 and 2020, with carbon emissions decreasing in the process of rapid economic development and achieving high-quality development. Examples show that this method provides an important reference for exploring sustainable development and high-quality development under the background of low-carbon economy. However, there are shortcomings in the improved ecological footprint. The limitation of statistical data does not allow for the visualization of small areas of carbon emissions, and future research and improvement of carbon detection can be used to accurately represent the spatial and temporal variation of the carbon footprint in small areas.

### Conclusion

This paper measures and spatially visualizes the ecological footprint of the Yellow River Delta for 2015, 2018 and 2020 with the help of a carbon footprint improvement ecological footprint model. Additionally, in the context of a low carbon economy, the decoupling index of carbon emissions and GDP is extended to the evaluation and analysis of high-quality development. In summary, the following conclusions are drawn.

(1) From 2015 to 2018, carbon emissions increased by 54.733 ten thousand tons, with an average annual increase of 18.244 ten thousand tons and a reduction in carbon sinks of approximately 0.06 ten thousand tons in the same phase; from 2018 to 2020, carbon emissions increased by 102.59 ten thousand tons, with an average annual increase of 51.295 ten thousand tons and an increase in carbon sinks of 3.612 ten thousand tons, a significant increase in carbon emissions compared to the first phase, with an increase of approximately twice as much as the first phase. Construction land is the main source of carbon emissions, accounting for more than 98% of the total carbon emissions in the same year. Therefore, the structure of land resources should be optimized, the scale of construction land should be strictly controlled, the utilization rate of construction land should be increased, and the energy utilization rate of energy-intensive industries should be improved.

(2) The ecological footprint per capita has been growing year by year, with the carbon footprint surpassing the ecological footprint of other biologically productive land, averaging 0.217 hm^2^·people^− 1^ per year, accounting for 28.2% of the total ecological footprint in the same period; the ecological carrying capacity per capita has decreased from 0.3953 hm^2^·people^− 1^ in 2015 to 0.3074 hm^2^·people^− 1^ in 2020; and the ecological deficit per capita is in a long-term growth state, rising from 0.3258 hm^2^·people^− 1^ in 2015 to 0.4501 hm^2^·people^− 1^ in 2020. From a county perspective, the total ecological footprint of Dongying District far exceeds that of other counties, and although its total ecological carrying capacity also far exceeds that of other counties, its overload level is at the top of all counties. Overall, the ecological pressure on the Yellow River Delta is increasing year by year and developing over time, and is generally in an ecologically unsustainable state.

(3) The areas with high ecological footprints are often located in the main urban areas of the Yellow River Delta and tend to spread outward from the center of the county, so the ecological footprints are mostly concentrated and located in Dongying District and Guangrao County, The main reason for this distribution is that Dongying District and Guangrao County have the highest GDP level among other counties and districts, have more land for construction, have a more mobile population and have strong aggregation. Second, the increase in construction land and the continuous development and use of cropland, unused land and coastal inland waters have created a spatial variation in ecological carrying capacity, with coastal areas as the main change. The ecological deficit is lightly overloaded in the urban core and moderately and heavily overloaded in the center of the core, where people tend to congregate, while the ecological surplus and ecological balance are mostly found in the northern and eastern parts of the Yellow River Delta where there is much arable land and inland water.

(4) In 2015 and 2017, the state of absolute decoupling was achieved. Economic development was not completely dependent on energy. With the rapid growth of GDP, carbon emissions decreased, and the level of high-quality development was high. At the same time, the ecological footprint and ecological deficit were at a low level in the overall research time sequence, and the ecological protection situation was good. Although it reached the absolute decoupling state in 2020, its ecological footprint and ecological deficit reached the peak of the research time sequence, and its ecological carrying capacity was relatively low, which led to an increase in ecological pressure and a sharp increase in carbon emissions. In the future, it is still possible to promote economic development at the expense of the ecological environment. Therefore, in the future, on the one hand, ecological protection should be the focus, rationally allocating land resources in the Yellow River Delta to protect the area of unique wetlands and reduce ecological pressure. On the other hand, carbon management should be strengthened and importance attached to the development model in a low-carbon economy, which will improve the system construction of a green economy. Ecological protection, economic development and low carbon land use should be effectively combined to achieve ecologically sustainable green high-quality development.

## Data Availability

The datasets used and analyzed during the current study are available from the corresponding author on reasonable request.
